# Functionalized multidimensional biomaterials for bone microenvironment engineering applications: Focus on osteoimmunomodulation

**DOI:** 10.3389/fbioe.2022.1023231

**Published:** 2022-11-04

**Authors:** Bin Lv, Juntao Wu, Yuan Xiong, Xudong Xie, Ze Lin, Bobin Mi, Guohui Liu

**Affiliations:** Department of Orthopaedics, Union Hospital, Tongji Medical College, Huazhong University of Science and Technology, Wuhan, China

**Keywords:** biomaterials, bone tissue engineering, osteoimmunomodulation, bone regeneration, drug delivery

## Abstract

As bone biology develops, it is gradually recognized that bone regeneration is a pathophysiological process that requires the simultaneous participation of multiple systems. With the introduction of osteoimmunology, the interplay between the immune system and the musculoskeletal diseases has been the conceptual framework for a thorough understanding of both systems and the advancement of osteoimmunomodulaty biomaterials. Various therapeutic strategies which include intervention of the surface characteristics or the local delivery systems with the incorporation of bioactive molecules have been applied to create an ideal bone microenvironment for bone tissue regeneration. Our review systematically summarized the current research that is being undertaken in the field of osteoimmunomodulaty bone biomaterials on a case-by-case basis, aiming to inspire more extensive research and promote clinical conversion.

## 1 Introduction

Bone is an organised, dynamic and functionalized organ, which supports fleshy structures, protects vital organs, and participates in various physiological functions. Bone is constantly and dynamically renewed in healthy individuals to maintain its mechanical properties. In the process of bone remodelling, a commensurate amount of new bone will replace the aged and/or damaged bone to maintain the mass and quality of normal bone. It is estimated that the number of fractures will increase by 28% from 2010 to 2025 annually in Europe, with an absolute increase from 3,500,000 to 4,500,000 injuries (Hernlund et al., 2013). Although bone trauma has the ability to heal by itself in normal condition, bone regeneration depends on the type of defect. Additionally, many factors including the age, metabolic status, and severity of the trauma can influence the probability of bone healing in these fractures. The most common form of endogenous bone healing consists of five phase, the last of which can be subdivided into three sequential stages: initiation, transition, and formation. Failure in bone healing will ultimately culminate in the suppression of blood supply to the tissue, which will result in the non-union of the bone due to ischemia, osteonecrosis, and bone loss (Buckwalter, 1996).

Focusing on the enormous potential in treating particular bone defects and the construction of biological substitutes for the injured bone, bone tissue engineering (BTE) has received widespread attention (Schuknecht, 1975). Efforts to repair bone can include the implantation of bone grafts and the development of synthetic permanent bone substitution grafts (Bueno and Glowacki, 2009). Initially, biomaterials are required to be non-toxic and biocompatible to the body. Moreover, with the insights into ‘Osteoimmunology’ and the ongoing development of BTE, more attention have been directed to the crosstalk between the immune system and the bone microenvironment. All the cells in the bone share the same microenvironments, which acts as an important role in osteogenesis. A suitable osteogenic microenvironment consisting of cellular and contextual components provides the basis for bone regeneration. Specifically, the cellular components include bone cells, immune cells, mesenchymal stem cells, and endothelial cells, while the contextual components mainly include cytokines, chemokines and growth factors (Song et al., 2020). Recently, osteoimmunomodulation has emerged as a promising principle in the design of bone biomaterials, which emphasizes the interactions between the immune and skeletal systems and contributes to tuning a beneficial osteoimmune microenvironment by inducing a suitable inflammatory response to enhance osteogenesis indirectly (Zhou and Groth, 2018). Consequently, various strategies, including the active construction of the local delivery system with the incorporation of bioactive molecules or passive intervention of the surface properties including physical and chemical characteristics, have been applied to create a favorable bone microenvironment. For one thing, the chemical composition, biophysical features, and surface properties of substrates influence mechanobiology of stem cells and nuclear organization, affecting attachment and migration of cells but also osteogenesis and matrix mineralization (Cun and Hosta-Rigau, 2020). For another, mimicking of the bone microenvironment is also described as affecting osteogenesis by virtue of the regulation of the surface chemistry and delivery of biomolecules through protein coating or presence of hydroxyapatite in the structures (Cheng et al., 2019).

In this review, the first section highlights the involvement of immune systems in bone regeneration and its potential application. The second section focuses on functionalized multidimensional biomaterials that modulate the bone immune microenvironment and exert a positive influence on bone regeneration. Finally, challenges and future directions of biomaterial-based strategies for osteoimmunomodulation are discussed.

## 2 Involvement of immune cells in bone regeneration

To design biomaterials with favorable osteoimmunomodulatory and regenerative properties, it is necessary to elucidate the main upstream effector immune cells and their functions throughout the whole regeneration process first, thus revealing the mechanism of activation of immune cells and the principle of osteoimmunomodulation. Among immune cells, macrophages are the dominant effector cells, functioning to maintain bone homeostasis and play a prominent role in bone repair. In most studies, the macrophage polarization from M1 to M2 is the key strategy for immunomodulation for bone regeneration. The interaction of immune cells can be used to better guide bone regeneration particularly between macrophages and T lymphocytes, thus revealing possible novel target points for osteoimmunomodulatory treatment strategies (Vishwakarma et al., 2016).

### 2.1 Macrophages

Macrophages are a type of phagocyte present in every organ, which is considered to be the organism’s first line of defense. Macrophages can be roughly classifeid into resident macrophages and inflammatory macrophages (Raggatt et al., 2014). Macrophages are key participants in the initial inflammatory response and the overall response to the implanted biomaterials, which determine whether there will be the formation of fibrous cysts or the regression of the inflammatory process, as well as the bone regeneration resulting therefrom. Therefore, it is necessary to understand the role of macrophages in bone regeneration and the regulatory role of biomaterials in the behavior of macrophages.

#### 2.1.1 Tissue-resident macrophages

Osteoclasts are considered to be resident macrophages in bone, and they are in close proximity to osteoblasts on the surface of bone endosteal cells, suggesting that bone macrophages may provide pro-growth support to osteoblasts and promote bone formation. They release proteolytic enzymes and acids and dissolve collagen and mineral bone matrix. Murine osteoclastogenesis is driven by osteoblasts producing RANKL and OPG, which is named the RANK-RANKL-OPG system (T'Jonck et al., 2018). In bone tissue, osteoblasts, osteocytes and immune cells express RANKL, with higher expression of RANKL in osteoblasts and osteocytes. The binding of RANKL to RANK promotes osteoclast differentiation by activating downstream cascade reactions. The elucidation of downstream signaling pathways of the RANKL–RANK axis in osteoclastogenesis revealed a number of shared molecules and signaling mechanisms between bone and immune cells. Many immune system factors affect the RANKL/RANK pathway and thus regulate bone regeneration. The cytokine binding domain of OPG binds RANKL with higher affinity and interferes with RANKL-RANK to further inhibit osteoclast formation, resulting in increased bone mass (Nelson et al., 2012). M-CSF released by osteoblasts acts as a potent stimulator of RANK when bound to c-Fms. DCs are able to differentiate into osteoclasts in trans through the RANK/RANKL pathway and interact with CD4+T cells. For example, B lymphocytes release OPG, while activated T and B lymphocytes can release RANKL. Neutrophils can also express RANKL to regulate osteoblasts and trigger bone resorption. Inflammatory cytokines indirectly affect bone conversion, such as TNF, which promotes osteoclastogenesis through RANK and RANKL. On the other hand, RANK/RANKL/OPG can be regulated by cytokines (e.g., IL-1, IL-6, IL-7, and IL-17A) (Weitzmann, 2017). IL-6 and IL-23 can increase RANKL expression, and OSM promotes osteoclastogenesis by stimulating the osteoblast production of RANKL in synergy with IL-6, which in turn affects bone metabolism (Abdel Meguid et al., 2013).

#### 2.1.2 Inflammatory macrophages

Inflammatory macrophages originate from monocytes and reach the site of inflammation through the bloodstream. Once activated, macrophages possess several phenotypes, which are responsive to environmental cues in a dynamic and plastic way (Mantovani et al., 2013). The polarization state of macrophages is unstable as they can easily switch between states according to the microenvironment (Liu et al., 2014). M1 macrophages are able to kill pathogens and accompany inflammation, exhibiting typical signs of chronic inflammation, whereas M2 macrophages have the ability to promote bone regeneration. M2 macrophages have been reported to promote osteoblast differentiation in MSCs and bone mineralization (Gong et al., 2016). β-TCP-stimulated macrophages promote osteoblast differentiation in bone marrow mesenchymal stem cells (Chen et al., 2013). All these results suggest a crucial role of macrophages in bone regeneration during skeletal inflammation.

**FIGURE 1:**
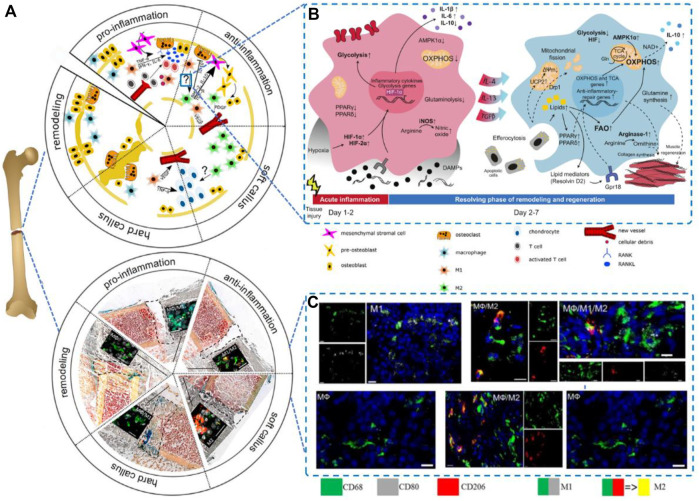
Macrophages in bone regeneration.**(A)** Five phases of the process Reproduced from (Schlundt et al., 2021); **(B)**Metabolic rearrangement in macrophage polarization to proinflammatory or alternatively activated macrophages *in vivo*; Reproduced from (Wculek et al., 2022); and **(C)**A switch from M1-to M2-polarized macrophages present in and around the injured side. Reproduced from (Schlundt et al., 2018).

The classical M1 macrophages activated by inflammation are able to produce high levels of inflammatory cytokines (e.g., IL-6, IL-1β, and TNF-α) and oxidative metabolites (e.g., nitric oxide and peroxides), which induce osteoclastogenesis. M2 macrophages can be significantly induced by IL-4, and activated M2 macrophages attenuate the inflammatory response and prevent TNF-α-mediated bone loss (Al-Maawi et al., 2017). They also promote osteogenesis in the presence of BMP-2 and VEGF. The transition from pro-to anti-inflammation in the fracture area is needed to enable the regeneration of the injured bone, which could be mediated by a switch of macrophage type from M1-to M2-in or around the injured site (Figures 1A,C). The macrophage phenotype can be dynamically and plasticly altered according to the local microenvironment, causing these cells to change their phenotype and physiological functions, whereby some studies have attempted to improve bone regeneration by regulating the number of macrophages or their polarization to the M1 and M2 phenotypes (Pajarinen et al., 2019). *In vivo*, macrophage polarization is regulated by a variety of cells and cytokines, and macrophages can be activated to the M1 type by the classical activation pathway mediated by γ-interferon and lipopolysaccharide or to the M2 type by the alternative pathway in response to factors such as IL-10 and IL-13 (Figures 1A,B).

### 2.2 T cells

**FIGURE 2:**
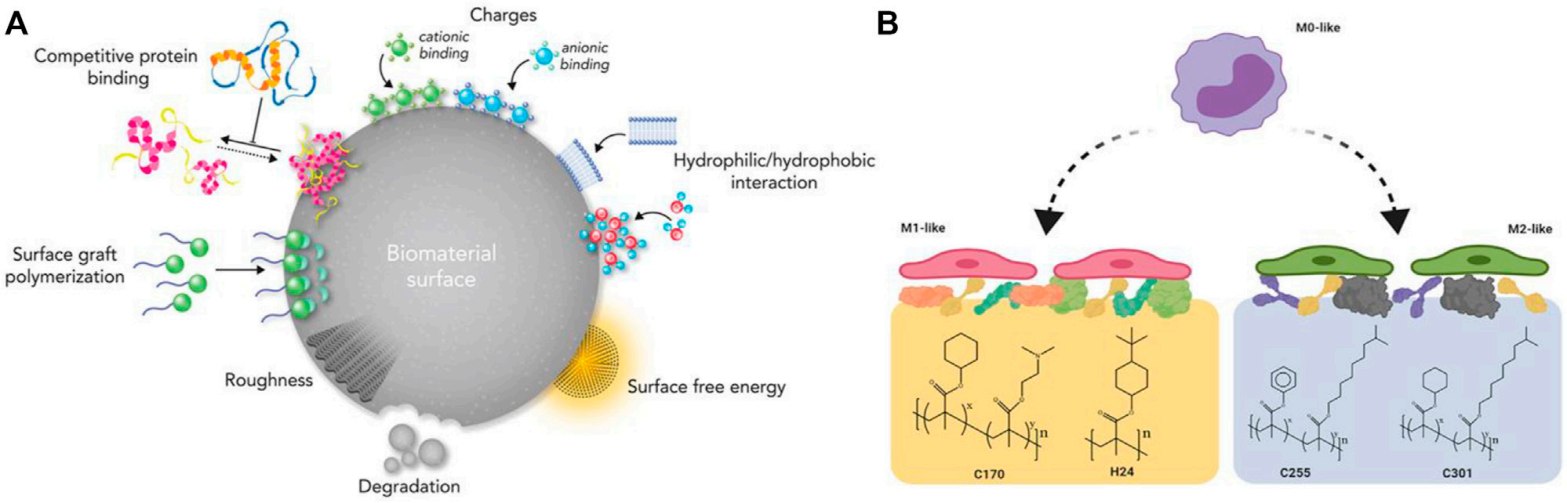
**(A)** An illustration of the surface physicochemical characteristics of biomaterials. Reproduced from (Rahmati et al., 2020); **(B)** Macrophage polarization using surface chemistry. Reproduced from (Rostam et al., 2020).

Previous studies have shown that the polarization of macrophages to the M1/M2 phenotype relies on T-cell involvement (Vasandan et al., 2016), which indicates that T cells are target cells in bone regeneration. T cells can be divided into CD4^+^ T cells and CD8^+^ T cells primarily. CD4^+^ T cells can be classified into different subsets according to their their cytokine expression profile (Th1, Th2, Th17, and Treg). Thus, it is generally considered that CD4^+^ T cells are a controlled object for biostimulation-induced bone regeneration. TNFα can stimulate osteoclastogenesis *via* mediating RANKL expression by macrophages (Lam et al., 2000). IL-17 can stimulate bone resorption *via* inducing osteoblast differentiation and the RANKL secretion by osteoblasts, (Lee, 2013). Apart from the traditional CD4^+^ T cells, it has been demonstrated that Treg can promote steogenic differentiation directly and enhance bone regeneration by inhibiting CD4^+^ T-cells secreting TNF-α and IFN-γ. Activated Th2 cells can secrete IL-4 which can induce macrophage polarization. Then polarized macrophages serve as an imperative factor in bone regeneration. To conclude, the aim to promoting bone repair is promising by manipulating adaptive immunity to create favorable immune microenvironment responses through BTE (Sadtler et al., 2016) (Figure 2).

## 3 Functionalization for osseointegration and osteoimmunomodulation

As mentioned above, ideal bone biomaterials can promote tissue regeneration in the defect area through immunomodulatory functions. Osteoimmunomodulatory biomaterials can systematically regulate cell behaviors (such as macrophages) and the osteoimmune environment, consequently affecting bone regeneration. Recently, the active construction of local drug delivery systems has been the main strategy for osteoimmunomodulation, which should conform to the basic princilples: 1) The drug can be dispersed in the scaffolds; 2) The drug can be released at a certain rate; 3)The scaffold materials can remain stable for a long time, while protecting the activity of drugs. For inorganic scaffolds, ionic components (e.g., Ca, Si, Mg, Zn) can be incorporated into the scaffolds. With the degradation and release of scaffolds, they can not only act on the cells attached to the bone-implant interface, but also play a role on the “remote” cells (Table 1).

**TABLE 1:** Mediators involved in the composition of delivery system for osteoimmunomodulation.

Class	Molecules	Biomaterials		Vivo model	Treatment outcome	References
Drug	Dexamethasone	aminated mesoporous silica nanoparticles (MSNs-NH2)	(PLLA/PCL) nanofibrous scaffold	Rat; adult male; calvarial bone defect	Enhanced osteogenic differentiation and mineralization	Qiu et al. (2016)
	Simvastatin	poloxamer 407 hydrogel	3D-printed porous titanium scaffolds (pTi scaffolds)	Rabbit; cylindrical defects (5 mm in diameter and 5 mm in depth)	Improved osseointegration, bone ingrowth and neovascularization	Liu et al. (2016)
	Salicylic acid	SA-based polymer scaffold		/(in mimic physiological conditions)	Prolonged mitigation of inflammation; Targeted diabetic bone regeneration	Yu et al. (2017)
	ibuprofen	HA particles		—	Improved bone-implant interlocking and enhancement for new bone formation	Safi et al. (2018)
Lipids	TGF-β1	PLA/PCL Scaffolds		—	Enhanced bone regeneration	Cheng et al. (2021)
	Maresin1	/(Systemic delivery)		Mouse, transverse fracture of the tibia	Improved bone healing; decreased pro-inflammatory macrophages; decreased circulating IL-1, IL-6, IL- 10, TNF-α, KC, MCP-1	Huang et al. (2020)
	Resolvin D1	Biomimetic Anti-inflammatory Nano-Capsule (BANC)	boron-containing meso-porous bioactive glass (B-MBG) scaffolds	Mouse; femoral defect (1 mm)	Enhanced M2 macrophage polarization; Enhanced the bone regeneration in the defect area	Yin et al. (2020)
Cytokines	IL-4	Decellularized bone matrix (DBM) reservoir		Rat; a defect in the center area on each calvaria (5 mm)	decreased production of TNF-α Enhanced osteogenesis and angiogenesis	Zheng et al. (2018)
		nanofibrous heparin-modified gelatin microsphere (NHG-MS)		Rat; induced Type II diabetes; mandibular periodontal fenestration defect	Recovered M2/M1 ratio; Enhanced osteoblastic differentiation; Restored bone regeneration	Hu et al. (2018)
	IL-4+IL-13	collagen scaffold		Mouse, male; unilateral closed fractures in the left femur	Improved bone regeneration; increased M2 macrophages	Schlundt et al. (2018)
	IL-4+SDF-1a	TG-gels		Rat; periodontal defect (2 × 2 mm)	Enhanced periodontal healing; increased M2 macrophages; increased MSCs	He et al. (2019)
	IL-4/RGD peptide	TiO2 nanotubes		—	Increased production of IL-10 Enhanced the differentiation of MSC	Li et al. (2020b)
Growth Factors	BMP-4	mesoporous silica nanoparticles (MSNs)	GelMA/gelatin/PEG scaffold	Rat male; 5 mm diameter calvarial defect	Promoted osteogenesis of BMSCs; Regulated M2 type macrophage polarization; Secreted BMP-2	Sun et al. (2020)
	VEGF + BMP-2	HMPs in alginate gel		Rat femoral injury model	Accelerated femoral healing and vascularization *in vivo*	Subbiah et al. (2020)
Purine	Adenosine	PEG-PBA macroporous scaffold		Mouse; transverse tibial fracture	Improved bone healing; increased angiogenesis	Zheng and Wang, (2020a)
		Aln-NC nanocarriers		Mouse; Athymic nude	promoted new bone formation; improved bone mechanical strength	Hoque et al. (2021)
	cAMP	HA/Gel scaffolds		Rat; calvarial defect (5 mm in diameter and 3 mm in depth)	Accelerated bone healing	Ju et al. (2021)
	ADORA2a	Fibrin gel		Rat; transverse tibial fracture, burnt periosteum	Accelerated bone healing	Zheng and Wang, (2020b)
	PGI2 analog	Biphasic fibrin		Mouse; femoral osteotomy (0.7 mm)	Improved bone healing; decreased CD8+IFNγ+ T cells; decreased M1 macrophages; increased M2 macrophages	Wendler et al. (2019)
Bioactive factors	Mg	Fibrinogen scaffold		—	Reduced LPS-induced TNF- α secretion; increased MSC ALP activity; Reduced macrophage pro-inflammatory stimulation	Bessa-Gonalves et al. (2020)
	Sr	Sr-loaded PTL coating	Ti	Rat; femora implant	Enhanced BMSCs recruitment and osteogenic differentiation; Improved bone formation	Lu et al. (2019)
	Sr Ag	AH-Sr-AgNPs	pure titanium (Cp‐Ti)	Rabbit; femoral metaphysis defect	Enhanced osteogenic outcome through favorable immunoregulation	Chen et al. (2019)
	Ga	PCL/MBG/Ga scaffold		Rabbit; radius defect (15 mm)	accelerated bone healing and prevented bone resorption	Wang et al. (2021b)

**FIGURE 3:**
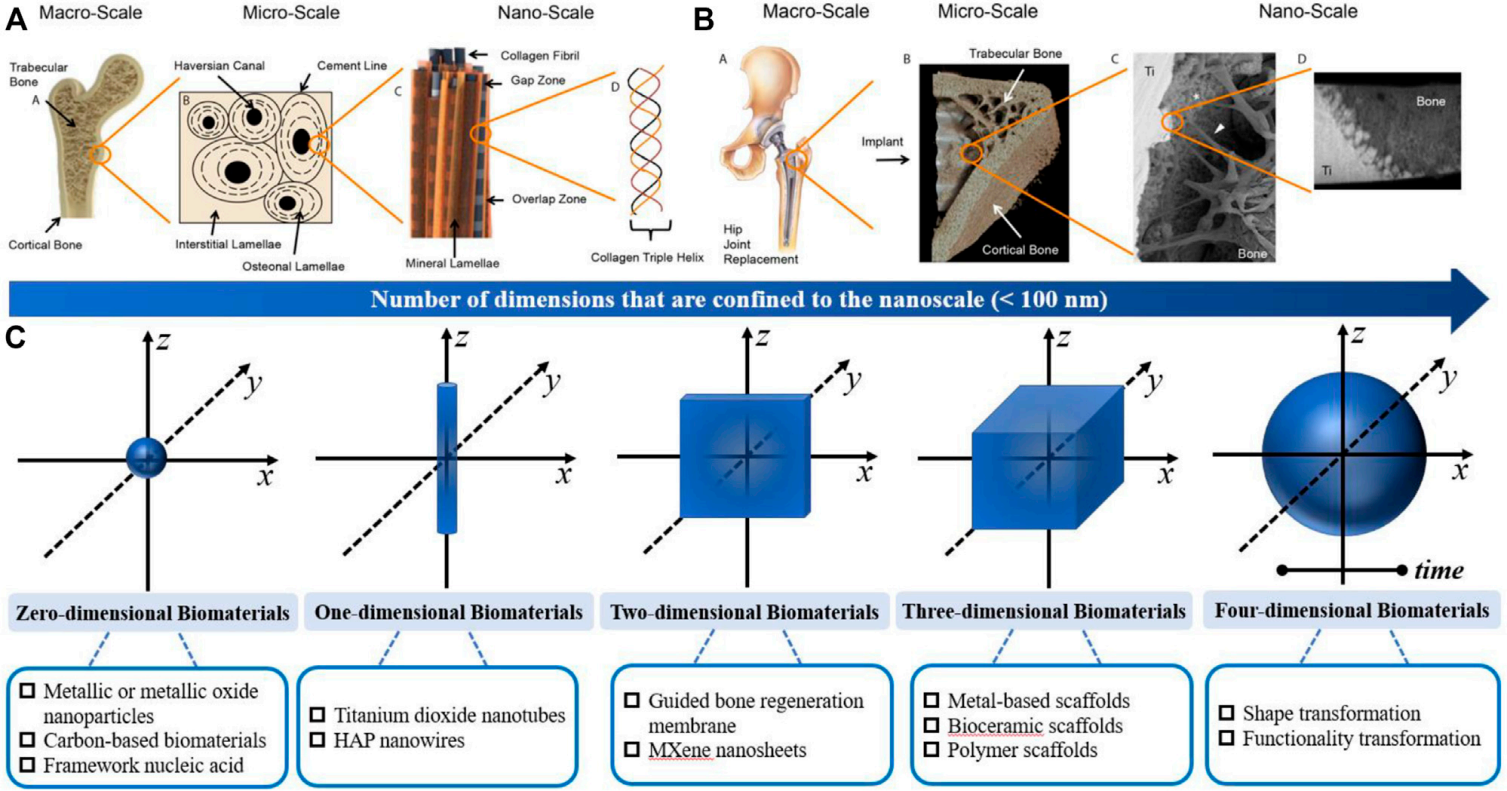
Structure of the bone and characterization of the osseointegration; **(A)** Hierarchical structure of bone from the macro-to nanoscale; **(B)** Hierarchical characterization of osseointegration from the macro-to nanoscale; Similar to bone, the connection at bone−implant interfaces spans several length scales. Reproduced from (Binkley and Grandfield, 2018); **(C)** Schematic overview of the functionalized multidimensional biomaterials from 0D-4D.

**FIGURE 4:**
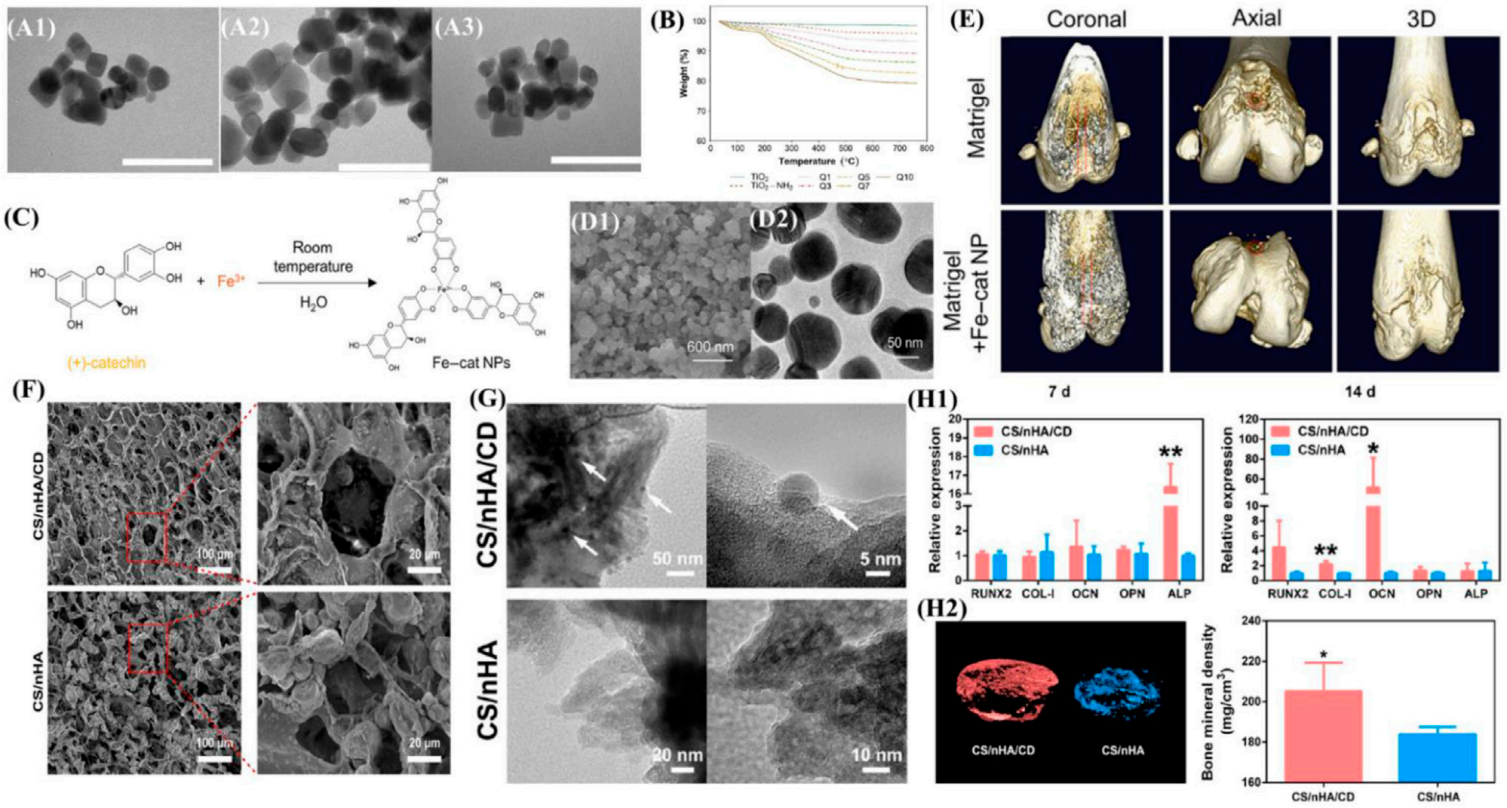
Overview of zero-dimensional biomaterials and evidence of their ability to promote bone formation. **(A)** TEM images of (A1) bare TiO2 nanoparticles, (A2) TiO2-NH2 nanoparticles and (A3) LbL-coated (Q10). The bar represents 100 nm. **(B)** Thermograms of different LbL-DEX-coated Ti-O-NH2 substrates. Reproduced from (Alotaibi et al., 2019) **(C)** Schematic illustration of the self-assembled Fe–cat NPs. **(D)** SEM image and TEM image of the Fe–cat NPs. **(E)** Representative coronal, axial, and 3D images of rat femurs in the Matrigel group and Matrigel + Fe–cat NP group, provided by micro-CT. Reproduced from (Kong et al., 2022) **(F)** SEM image of the CS/nHA/CD and CS/nHA scaffolds. **(G)** TEM image of the CS/nHA/CD and CS/nHA scaffolds. The white arrows show the CD. **(H1)** Relative expression of osteogenesis-related genes after 7 and 14 days of culture. **(H2)** Micro-CT 3D reconstruction models of the newly formed bone in the scaffolds. More new bone formation was found in CS/nHA/CD scaffolds at 4 weeks. Reproduced from (Lu et al., 2018).

Moreover, the passive intervention of physical or chemical characteristics is also an important approach (Figure 3A; Table 2). The success of osseointegrated biomaterials is usually decided by the functional bone-implant interface (Figures 4A,B). Biophysical cues, which include morphology and topography, are indirect signals that can be transmitted *via* integrins. These signals are increasingly recognized as key regulators of the bone-implant interface, thus manipulating cell fate and influencing tissue regeneration. However, the specific mechanism is still unclear, which may be that it affects the adsorption of extracellular matrix proteins. Furthermore, high-throughput screening (HTS) method has been applied to study the relationship between the topography of materials with different micro patterns and macrophage adhesion and polarization status. The results indicates that the column with a diameter of 5–10 μm is the key to drive the adhesion of macrophages, and the density of the column with a diameter of 10 μm is the key to control the immune response (Rostam et al., 2020). In addition to biophysical cues, cells can also respond positively to biochemical signals on biomaterials (Figure 3B). Typically, different chemical functional groups on biomaterials influence surface properties such as wettability, solubility, reactivity, and charge. Thus, chemical modifications of biomaterials offer the potential to modulate cellular behavior. Taking macrophages as an example, it is found that hydrophilic surfaces showed more potential to promote macrophage differentiation into an anti-inflammatory phenotype, which indicate that macrophage polarization responds to different topographies and surface roughness differently (Lv et al., 2018).

**TABLE 2:** Key Physicochemical characteristics affecting the immune reactions to the implanted biomaterials.

Surface characteristics		Inpacts on immune cells		
Physical characteristics	Roughness	evoks immune responses significantly	
		Exerts an impact the cell adhesion	
	Particle size	unclear	
	Porosity and pore size	bigger pore size:decreased inflammation; enhanced angiogenesis	
Chemical characteristics	Wettability	hydrophobicity	increased monocyte adhesion
		hydrophilicity	decreased macrophage adhesion
	Charge	anionic/neutral particles	decreased inflammatotion
		cationic species	enhanced inflammation

## 4 Different categories of osteoimmunomodulatory biomaterials

Usually, biomaterials are classified according to their chemical composition or the response to the body. However, considering the dimensional geometry of biomaterials, we categorized osteoimmunomodulatory biomaterials based on the dimensional geometry in this article [i.e., the size of biomaterials in each dimension]. In general, the nanoscale is defined as 0.1 nm–100 nm. Particularly, 0D bone biomaterials have three dimensions strictly confined to the nanoscale, such as nanoparticles; 1D bone biomaterials have two dimensions confined to the nanoscale, such as nanotubes; 2D bone biomaterials have only one dimension within the nanoscale; and 3D biomaterials are larger than the nanoscale in all dimensions (Nersisyan et al., 2017a). In recent years, 4D biomaterials integrate the concept of time as the fourth dimension (Tibbits, 2014). Herein, we summarize some osteoimmunomodulatory biomaterials from 0D-4D (Figure 3C). It is worth mentioning that the materials in each dimension are not completely isolated. For example, many study reported 3D scaffold incorporated with 0D-2D biomaterials to form multidimensional composite scaffolds for their specific osteoimmunomodulatory effects.

### 4.1 Zero-dimensional biomaterials

Nanoparticles with all three dimensions strictly confined to the nanoscale are defined as zero-dimensional biomaterials (Nersisyan et al., 2017b). Due to the high surface-to-volume ratio, zero-dimensional biomaterials exhibit several distinct physicochemical properties.

#### 4.1.1 Metallic or metallic oxide nanoparticles

Various inorganic nanoparticles have shown incredible potential for promoting MSC proliferation as well as facilitating the process of osteogenic differentiation and biomineralization. Alotaibi et al. (2019) functionalized titanium oxide nanoparticles with amino groups (Ti-O-NH2) *via* silanation in toluene and then developed a dexamethasone (DEX) coating on the surface. The drug coating achieved a sustained release of dexamethasone for up to several months, released DEX modulated the macrophage M2 type, reduced TNF-α and IL-6 production, suppressed local immune responses, and promoted osteoblast and fibroblast growth. (Figures 4A,B). Moreover, based on the concept of endocytosis of nanoparticles, the synthesis of metal–organic coordination complexes is an effective way to transfer soluble organic molecules into condensed nanoparticles (Ma et al., 2019), which can improve utilization. Kong et al. (Kong et al., 2022) synthesized Fe–cat NPs *via* a facile one-pot strategy, which can achieve pH-responsive intracellular delivery of catechin. The synergy of catechin molecules offers Fe–cat NPs multiple biological functions, including osteogenic and anti-inflammatory effects, and regulates macrophage M2 polarization to create a favorable osteoimmunomodulatory microenvironment for bone regeneration. (Figures 4C–E).

#### 4.1.2 Carbon-based biomaterials

Apart from inorganic nanoparticles, numerous carbon-based biomaterials including nanodiamonds (NDs) and carbon dots (CDs) have been fabricated. NDs is a new member of carbon nanomaterials, with 5–8 nm in the diameter and a high surface volume ratio. A variety of fascinating features of NDs (i.e., superior mechanical strength, excellent surface reactivity, and strong intrinsic fluorescence) make them promising bone biomaterials. The surface functionalization of NDs can mainly improve safety and biocompatibility and reduce toxicity. After evaluating the physicochemical properties of nanofibrous membranes, the addition of NDs into PLGA scaffolds remarkably promoted their mechanical performance in rupture tests, and the composite scaffold also exhibited great biocompatibility to enable the proliferation of MG-63 cells without stimulating considerable inflammatory reactions of RAW 264.7 macrophages (Parizek et al., 2012). One study demonstrated that modifying PLA-PCL scaffolds with NDs does not aggravate the tissue response in a subcutaneous implantation model and that the mode of physisorbed BMP-2 delivery shows attenuation of inflammatory responses (Suliman et al., 2016). CDs have also drawn widespread attention since emergence. CDs have great biocompatibility with minimal cytotoxicity when applied at appropriate concentrations (i.e., 10 μg/ml) (Han et al., 2019), while high concentrations of CDs (i.e., >50 μg/ml) could exert an inhibitory effect on cell proliferation (Geng et al., 2018). Lu et al. (2018) demonstrated that 0D CDs can enhance the potential of bone repair scaffolds to induce the osteogenesis and that CD-doped scaffolds have promising application in PTT for tumors and infections. (Figures 4F–H).

#### 4.1.3 Framework nucleic acid

As DNA nanotechnology develops, various FNA nanostructures featuring single molecular weight, well-defined structure, controllable size and shape, and other properties are constructed, which provide advanced tools for the application of nanomaterials in BTE. Meanwhile, the powerful cell-entry capacity and editable properties of FNAs offer great possibilities for targeted delivery and controlled release of growth factors and drugs during bone regeneration. One of the most stable framework structure models is the tetrahedral framework nucleic acid (tFNA), in which four specific single-stranded DNAs self-assemble by complementary base pairing. The structure and synthesis of tFNA is very simple, with good structural stability and biological activity (Li et al., 2019; Lin et al., 2020). Zhao et al.(2020) constructed a 200 bp tFNA, formed by 4 ss-DNAs with a specific base sequence, which can promote angiogenesis and M2 polarization in macrophages and promote the treatment of BRONJ both *in vitro* and *in vivo*.

### 4.2 One-dimensional biomaterials

1D biomaterials whose only two dimensions are confined to the nanoscale (<100 nm), can be classified into nanotubes, nanowires, *etc.* (Nersisyan et al., 2017a). Owing to the unique morphology (e.g., high length-to-diameter ratio) and nanotopography, 1D biomaterials have an extremely high degree of anisotropy, which results in various distinct properties. In addition, many 1D biomaterials have become the basic building blocks for the fabrication of higher-dimensional biomaterials.

#### 4.2.1 Carbon nanotubes

CNTs, as a novel 1D material, is one of the current research hotspots, which possess the following advantages in the bone repair: 1) CNTs have excellent mechanical properties; 2) The large surface area and excellent conductivity of CNTs are more conducive to protein absorption and cell adhesion growth (Mirmusavi et al., 2019); 3) CNTs can be introduced into the matrix materials as reinforcement materials to obtain the structure of nano-network and appropriate porosity, which is more favorable to the material exchange of extracellular matrix in the bone tissue (Holmes et al., 2016). Du et al. (2021) found that CNTs can promote the polarization of M2 macrophages, suggesting that CNTs may play a role in this process. In addition, the large specific surface area and hollow structure of CNTs endow them with strong capacity of drug carrying, and can make adjustment to the release of drug, thus improving the permeability and retention of drug. Sukhodub et al. (2018) used the composite containing CNTs to carry dexamethasone, and found that it has the effect of inducing bone formation.

#### 4.2.2 Titanium dioxide nanotubes

**FIGURE 5:**
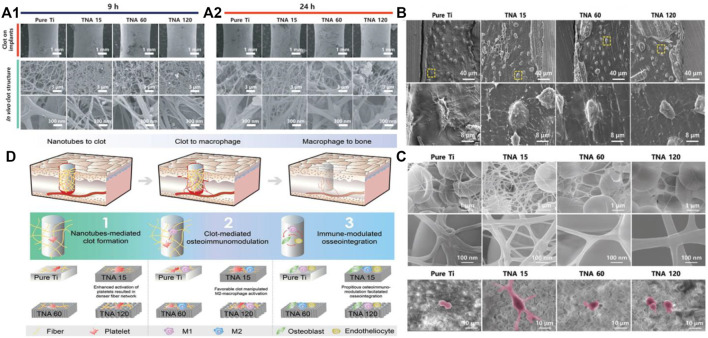
**(A)** Macroscopic images of clot formation after implantation *in vivo*; **(B)** SEM images of the osteocytes-to-implant interface. **(C)** SEM images of the clot and THE MΦ (purple area) after coculturing; **(D)** The connection between the TiO_2_-tubes, clot and osteoimmunomodulation.

In Bai et al. study, TiO_2_-NTs with different diameters are fabricated on Ti and results show that surface nano size can significantly affect thrombosis, and appropriate thrombus characteristics can manipulate favorable bone immune regulatory environment to promote bone regeneration and integration (Figure 5). In addition, TiO_2_-NTs can serve as an excellent delivery platform for inorganic bioactive elements (Mg, Zn and Sr), drugs and growth factors. Li et al. (2020a) made titanium oxide nanotubes (TNT) with excellent biocompatibility capabilities by electrochemical anodization, which possess a high surface area for drug loading and long-term drug elution into implants. Moreover, numerous methods including structural changes in the diameter and length, the use of biodegradable polymer coatings, and the application of polymer micelles as carrier nanoparticles have been used to control the release of delivery better (Sinn and Losic, 2012). In this work, bioactive Mg-doped TiO2 NTs were generated on Ti implants by anodic oxidation and hydrothermal treatment. The results show that the MgN coating of titanium can influence the polarization of macrophages and induce a favorable immune environment for osteogenesis. Controlling the concentration of Mg ions in Mg-based bone scaffolds gives the biomaterials good bone immunomodulatory properties, thus providing essential evidence for improving and modifying the effects of Mg-based bone biomaterials. It was found that trace amounts of magnesium ions (100 mg/L) s inhibit the TLR-NF-κB signaling pathway and induce M2 phenotypic changes and releasing anti-inflammatory cytokines in macrophages, while magnesium ion/macrophage conditioning mediators expedited the process of osteogenesis in BMSCs through the BMP/SMAD signaling pathway (Zhang et al., 2019a).

#### 4.2.3 HAP nanowires

HAP, the major inorganic component of bone, has an irreplaceable role in the bone regeneration. Ultralong HAP nanowires exhibit high flexibility and could interweave with each other compared with brittle HAP materials. By simulating the structure of natural cancellous bone, Huang et al. (2021a) assembled ultralong HAP nanowires into HAP nanowire aerogel with highly efficient filtration and good elastic aerogel by freeze-drying method. Moreover, the application of the single-phase HAP nanowire aerogel has been systematically investigated. At 12 weeks after implantation, HAP nanowire aerogel scaffolds significantly promoted the adhesion, proliferation, and migration of rBMSCs and the new bone growth of bone defect areas. In future studies, the incorporation of other functional components or loading of other growth-promoting factors into HAP nanowire aerogels may have further clinical therapeutic effects. Therefore, HAP nanowire aerogels have promising applications in bone defect repair.

### 4.3 Two-dimensional biomaterials

The concept of 2D biomaterial is based on the fact that only one dimension of it is within the nanoscale range (Nersisyan et al., 2017a). According to the definition, 2D biomaterials possess a large surface-to-volume ratio, good mechanical strength and ultrathin structure, which can bear loading a great amount of functional biomolecules. In addition, the large surface-to-volume ratio endows them with great potential to modify the surface, such as the chemical properties and charge, subsequently influencing cell fate. A large amount of 2D nanofilm coatings have been widely applied to facilitate more intense integration of biomaterials and the biological environment and enhance the bone regeneration.

#### 4.3.1 Guided bone regeneration membrane

**FIGURE 6:**
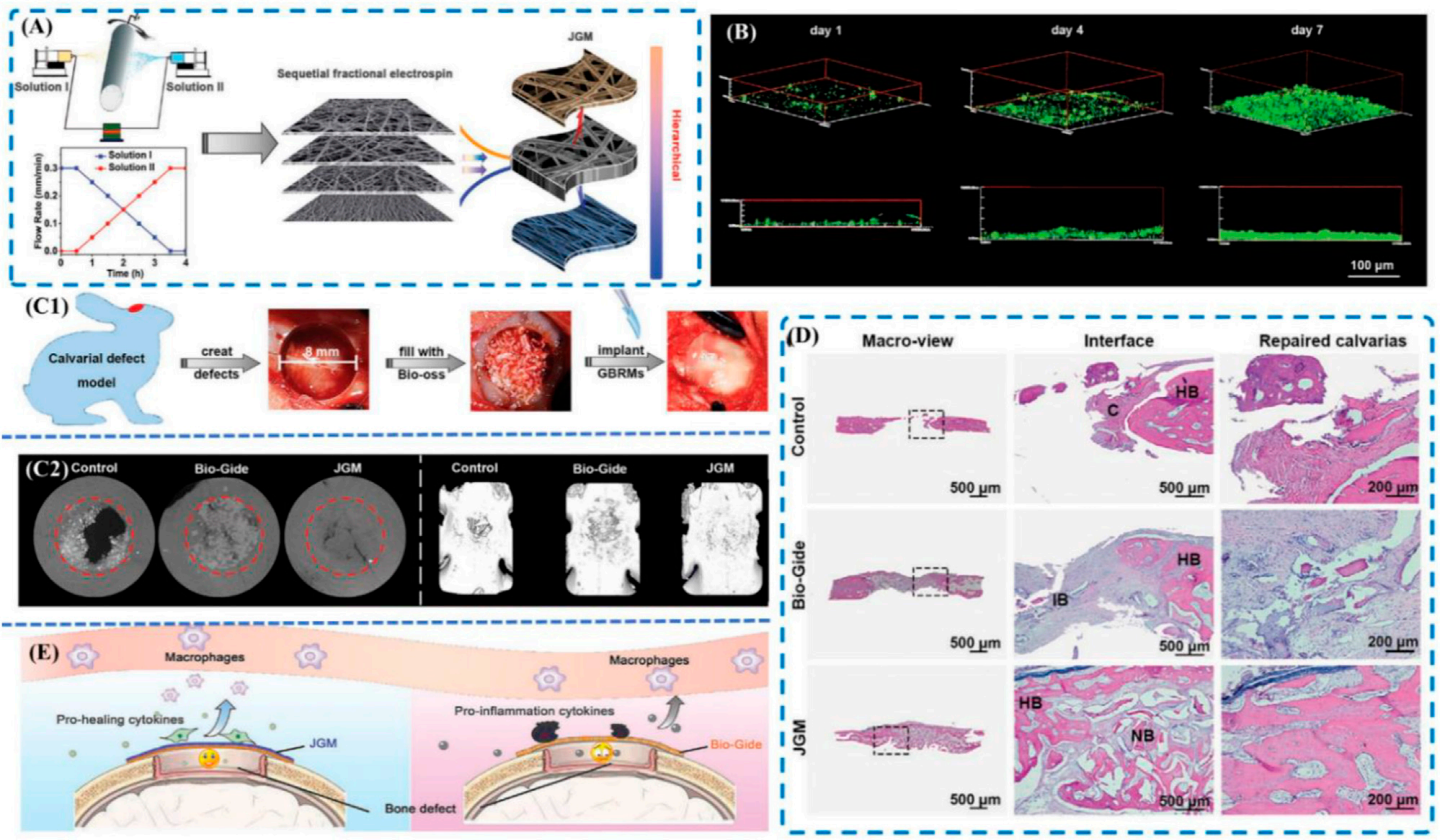
**(A)** Schematic of the sequential fractional electrospinning process; **(B)** 3D fluorescence images of MC3T3-E1 cells cultured on the inner face of the JGM for 1 day, 4 days, and 7 days; **(C)** *In vivo* bone repair evaluation. (C1) Schematic of the operation; (C2) Micro-CT images of the bone; **(D)** Histological analyses of calvarial defects at 8 weeks. **(A)** H&E staining images; **(E)** Schematic of the bone healing mechanism. Reproduced from (Chen et al., 2018).

With the gradual maturity of GBR technology, more and more non absorbable membranes used in clinical applications are gradually transformed into absorbable membranes mainly from type I and type III collagen of cattle and pigs (Barbeck et al., 2015). Chen et al. coated collagen membranes with nanometer-sized bioactive glass Ca2ZnSi2O7 to tune the osteoimmune environment successfully (Chen et al., 2018). Lately, the concept of Janus based on the idea that one can asymmetrically tailor the morphology structure, composition and bioactivity of each face to optimize the overall performance of the membranes has been widely applied. Wang et al. (2021a) designed a novel Janus guided bone regeneration membrane (JGM) fabricated by sequential fractional electrospinning. In this study, the fate of macrophages and hBMSCs was hypothesized to be orchestrated by the aligned topology and the improved surface wettability of the outer face of the JGM (Figure 6). Moreover, Arafat Kabir et al. (2021) demonstrated that the human concentrated growth factor (CGF) membrane could act as a biological delivery platform of BMP-2 and CGF/BMP-2 might become a bone reservoir on the periosteum of the skull for bone autografts.

#### 4.3.2 MXene nanosheets

MXene nanosheets (NSs) are representative 2D materials composed of carbides, nitrides or carbonitrides (Lin et al., 2017). At present, Ti3C2Tx is one of the most widely studied MXene, which was chosen to research the potential application of MXenes in bone regeneration (Zheng et al., 2016; Sinha et al., 2018). Fu et al. (2021) successfully prepared UHAPNWs/MXene nanocomposite membranes by coblending self-assembly and vacuum-assisted filtration. More importantly, among the MXenes with different weight ratios of UHAPNWs, those with 10 wt% prevents the highest tensile modulus and strength. It is indicated that the UHAPNWs/MXene can have a better preservation of osteogenic space and promote the bone regeneration further while the multilayered MXene film can direct osteoblast attachment and prevent non-osteogenic tissue from interfering with bone regeneration. Moreover, niobium carbide (Nb_2_C) MXene NSs are highly bio-compatible and biodegradable with intrinsic photoresponse in the second near-infrared (NIR-II) biological window for theranostic nanomedicine (Han et al., 2018). Yin et al. (2021) reported a rational integration of photonic-responsive Nb_2_C NSs into 3D-printed bone-mimetic scaffolds (NBGS) for osteosarcoma treatment.

### 4.4 Three-dimensional biomaterials

Most clinically used implants are three-dimensional biomaterials, i.e., biomaterials where all dimensions are larger than the nanoscale. The most investigated 3D biomaterials in the field of bone regeneration include metal scaffolds, bioceramic scaffolds, polymer scaffolds, and hydrogels. 3D biomaterials have a tunable spatial structure and biochemical properties and can be used as a simulated ECM to modulate cell behavior and determine cell fate.

#### 4.4.1 Metal-based scaffolds

Ti, Mg, Ta, and their alloys, as typical representatives of metal materials, are more mature in their clinical applications. For example, Ti and its alloy Ti-6Al-4 V are the most commonly used load-bearing metal implant materials with high corrosion resistance, enhanced mechanical properties, high strength-to-weight ratio and excellent osseointegration ability (Long and Rack, 1998; Geetha et al., 2009; Fu et al., 2011; Skorb and Andreeva, 2013). However, due to the lack of bioactivity and osteoconductivity, Ti and its alloys require at least 3 months after implantation to achieve adequate fixation (Albrektsson et al., 1986; Oshida et al., 2010).

Surface bioactive ion chemical treatment and nanoscale topographical modification are considered promising approaches to the current surface design of titanium implants. Hotchkiss et al. (2018) implanted titanium implants with different roughness surfaces into the femurs of male C57BL/6 mice and showed that macrophages can regulate MSC recruitment and the Th cell phenotype to generate an immune microenvironment for wound healing by releasing multiple cytokines in response to implant surfaces with different roughness and hydrophobicity. In addition, functionalization of surfaces by bioactive chemical elements might accelerate osseointegration. Lee et al. (2016) demonstrated that functionalization of surfaces by divalent cations (Ca and Sr) greatly induces the M2 regenerative phenotype of J774. A1 cells line in nanostructured surfaces. Moreover, Yang. (2012) proposed a stable and biomolecule-binding modification material, phase transition lysozyme (PTL), which can impart functional groups and positive charges through introduction of bioactive molecules and layer-by-layer self-assembly (Lv et al., 2014; Zhong et al., 2016; Ha et al., 2018). Lu et al. (2019) successfully integrated Sr into PTL coatings on Ti surfaces, demonstrating that the direct sustained *in situ* release of Sr2+ at the implant-tissue interface could dampen inflammation and facilitate implant osseointegration, and that Sr-containing PTL coatings modulate the behavior of BMSCs, enhance osteogenic differentiation, and stimulate greater osteogenesis by modulating the osteoimmunomodulation microenvironment. In addition, studies have shown that functionalization can improve the interface with high bond strength, such as the polymeric layer of polydopamine (PDA) rich in catechol groups, which can immobilize primary amine or thiol biomolecules through a simple impregnation process and provide secondary reaction for the coating material or incorporation of bioactive molecules (Madhurakkat Perikamana et al., 2015; Pan et al., 2016; Yang et al., 2018). Therefore, it is important to promote bone regeneration if the coating has universal adhesion properties and intelligent delivery properties (Qiu et al., 2019; Huang et al., 2021b), thus obtaining favorable osteogenic activity and immunomodulatory effects and promoting the interaction between the host and implant.

In natural ECM, there are microporous structures filled with water and soluble factors. The ECM-like structure offers mechanical support for cell and allows efficient transport of nutrients and wastes. Thus, the embedded nanoparticles exert biological effects. Porous titanium (PT) is generally considered as possessing favorable osteogenic capacity and can promote bone ingrowth because of its rationally designed porous structure and low elastic modulus (Charbonnier et al., 2021). Wang et al. (2022a) developed and systematically investigated a hierarchical biomodification of 3D-printed porous Ti6Al4V scaffold with macro/micro/nanoscales. Pan et al. (Zhai et al. , 2019) demonstrated that hierarchical macropore/nanosurface of pure Ti were equipped with tension-mediated immunomodulatory properties. The hierarchical macropore/nanosurface led to increased cytoskeleton tension which switched the phenotype of macrophages towards M2 and regulated the expression of inflammatory-associated genes. Meanwhile, the expression of BMP-2 and VEGF gene was also upregulated, causing the osteogenic differentiation and angiogenesis.

#### 4.4.2 Bioceramic scaffolds

With favorable printability, surface reactivity, mechanical properties, biological properties and cost-effectiveness, 3D printing bioceramic scaffolds have been widely used in clinical orthopedics (Dorozhkin, 2010; Ebrahimi et al., 2017; Wen et al., 2018). The osteogenic capacity of bioceramics can be attributed to the surface of scaffolds, which absorb osteoinductive factors or ions and release them into the microenvironment, and facilitate cell differentiation (Asa’ad et al., 20162016). However, the fatigue resistance and brittleness of bioceramics may deteriorate with increasing porosity, which limit their utility as load-bearing scaffolds (Dorozhkin, 2010). According to chemical bonds formed between implanted bioceramics and tissues, bioceramics are classified to bioceramics (e.g., calcium phosphate, bioactive glass, calcium sulfate, and calcium silicate) and bioinert bioceramics (Wen et al., 2018).

#### 4.4.3 Calcium phosphate

Calcium phosphates (CaPs) include hydroxyapatite (HAP), amorphous calcium phosphates (ACPs), biphasic calcium phosphates (BCPs), and dicalcium phosphate (DCP), octacalcium phosphate (OCP), and tricalcium phosphate (TCP) (Ebrahimi et al., 2017).

The CaPs can be fabricated in various forms, including coating layers, powders, granules, and bulk with tunable density and porosity. Considering that physical morphology has the characteristics of high controllability and stability, changing physical morphology to interfere with immune response has good application prospects. T'Jonck et al. (2018) compared the osteogenic ability of two CaPs with different surface morphologies; the submicron surface morphology caused bone formation, whereas no bone formation was observed for the micron surface morphology. Further studies showed that the submicron surface morphology recruited more macrophages and directed macrophage polarization through the PI3K/AKT pathway, confirming that material surface topography features can influence macrophage behavior by altering cell adhesion and cell shape.

Studies have shown that CaPs have immunomodulatory properties. Wang et al. (2017a) found that BCP particles and their degradation products augmented inflammatory cytokines secretion (e.g., IL-1, IL-6, monocyte chemokine 1, *etc.*) and growth factors (platelet-derived growth factor, VEGF, *etc.*) by macrophages. Similarly, CaPs ceramics can lead to infiltration of macrophages into the material implantation area, which in turn induces homing of bone marrow MSCs and ectopic osteogenesis (Wang et al., 2018). Moreover, Zarins et al. (2018) showed that strontium loaded on BCP particles significantly upregulated cytokines such as osteogenic protein 2/4 and IL-1, and exhibited excellent anti-inflammatory and osteogenic effects. The high expression of IL-1 accelerates the bone remodelling process in the region of local injury and affects the fracture healing process by promoting the proliferation of osteoblasts (Lange et al., 2010).

β-TCP can induce the polarization of macrophages into M2 type, and promote osteoblastic differentiation of bone marrow MSCs by up-regulating the expression level of WNT6 and down-regulating the expression level of WIF1 in Wnt signaling pathway (Zheng et al., 2021).

ZHANG et al. proposed that BCP can continuously release Ca2+, thus maintaining the long-term induction of macrophage polarization and promoting the differentiation of MSCs into osteoblasts (Zhang et al., 2021).

#### 4.4.4 Bioactive glass (BG)

The typical composition of bioactive glass (BG) is SiO2–Na2O–CaO–P2O5. According to the main component present in the composition, BG can be subdivided into three groups, namely, silicate (SiO2) glass, borate (B2O3) glass, and phosphate (P2O5) glass (Baino and Vitale-Brovarone, 2011). Taking advantage of the modifiability of BG components, BG can be functionalized, i.e., ions with anti-inflammatory, osteo- and angiogenic, and osteo-immune modulating effects can be released during BG degradation. Zhao et al. (2018) prepared strontium-containing bioactive glass microspheres (Sr-BGM) to promote angiogenesis by modulating the macrophage phenotype, and *in vitro* stimulation of macrophages with Sr-BGM induced their polarization toward the M2 type and induced them to express high levels of PDGF-BB. In addition, macrophage-conditioned medium with Sr-BGM significantly enhanced the angiogenic capacity of vascular endothelial cells (Zhao et al., 2018). Ebrahimi et al. (2017) prepared a porous Sr-BG scaffold that achieved near-perfect integration with host bone, and the morphology of the new bone tissue was almost close to that of normal bone. Numerous other ions, such as silver, copper, zinc, and lithium, are released with glass degradation to perform their corresponding biological functions when doped into BG (Baino et al., 2018). Although BG multifunctionalization can be achieved by incorporating different functional ions into BG, the addition of functional ions will certainly change the bioactivity, biocompatibility, and mechanical properties of BG. Even for multifunctional BG, the functional strength is often positively correlated with the amount of ions incorporated, and too large a proportion of incorporation and excessive accumulation of ions upon release can cause toxic effects on humans (Baino et al., 2018).

#### 4.4.5 Polymer scaffolds

Biocompatible polymers are polymeric compounds that posseess excellent biocompatibility and design flexibility. The degradability of polymer scaffolds is determined by composition and porosity (Liu and Ma, 2004). The following characteristics of polymer scaffold can be modified for tissue engineering: porosity (pore size and pore interconnectivity), biocompatible and bioactive, mechanically stable, biodegradability, mechanical properties (tensile strength and elasticity), surface functionalization and topographical clues.

#### 4.4.6 Natural polymers

Natural polymers originates from plants or animals, such as collagen, fibrin, gelatin, sodium alginate, cellulose, hyaluronic acid, and chitosan, are widely used in bone tissue engineering (BTE) because of their excellent biocompatibility and minimal negative immune effects. In addition, natural polymers may contain some biological recognition sites, which would achieve specific interactions with cells through functionalization, thus regulating cellular behavior (Hench and Polak, 2002). For example, in mice, covalent cross-linking of IL-1R1/MyD88 signal transduction inhibitor with fibrin matrix was able to antagonize the pro-inflammatory effect of IL-1β, differentiate MSCs toward osteoblasts, and promote bone regeneration (Martino et al., 2016). TP508 is a synthetic 23-amino acid peptide, and adsorbed fibrinogen made from this material promotes bone repair by inducing inflammatory mediator release and angiogenesis in a rat femur fracture model for bone repair and improved osteogenesis of bone defects in rats (Vasconcelos et al., 2016). Hyaluronic acid (HA), the main component of the ECM, can be employed to fabricate scaffolds, regulating tissue injury and accelerating the repair process (Knopf-Marques et al., 2016; Trombino et al., 2019). It is worth mentioning that the immunomodulatory effects of HA are strongly associated with molecular weight. High molecular weight HA has significant anti-inflammatory effects and induces IL-10 production by macrophages, whereas impaired low molecular weight HA promotes macrophage polarization toward the inflammatory phenotype and stimulates TNFa expression (Rayahin et al., 2015).

Synthetic Polymers

Synthetic polymers, include PLA, PGA, PLGA, PEG, PCL, etc, are semi-crystalline or amorphous (Mokarram and Bellamkonda, 2014). Compared with natural polymers, synthetic polymers are replicable and can be more easily tailored in terms of porosity, mechanical property, hydrophilicity, and degradability (Seal et al., 2001; Chen and Wu, 2005). Advanced fabrication techniques, such as electrospinning and rapid prototyping, have been extensively applied in the construction of interconnected porous microstructure of scaffolds. For example, Liu et al. (2021a) used electrostatic spinning to make fibrous membranes by adding DMOG and nSi to PLGA and then implanted them into the periodontium of rats. The results showed a decrease in CD40L and cd11b-positive cells and an increase in cd206-positive M2 macrophages, i.e., the immune response was directed toward promoting periodontal bone regeneration. However, PLGA can also be incorporated with other materials (e.g., ceramics, bioactive glass, or gelatin) to obtain favorable features due to the low the mechanical strength and poor osteoinductivity (Pan and Ding, 2012; Wang et al., 2017b). Studies have shown that the functionalization of PLGA combined with 10% doxycycline and 1% alendronate accelerates bone repair compared to using only drug-loaded PLGA (Limirio et al., 2016).

Surface modification of scaffolds with biomolecules (e.g., plasma deposition, Arg–Gly–Asp tripeptide (RGD)), could substantially ameliorate greater cell attachment and colonization (Yamaoka et al., 1999; Intranuovo et al., 2014). In addition, the synthetic peptide RGD tripeptide has been shown to facilitate the adhesion of myeloid cells to ECMs, inducing the macrophage to switch towards the anti-inflammatory phenotype through the action of integrins (Zaveri et al., 2014).

Natural polymers, synthetic polymers, and mixture nanofibers can be made into hybrid scaffolds. Compared with metallic materials, natural and synthetic hybrid scaffolds with high porosity display inferior stress-bearing capacity. For instance, Sheikh et al. (2016) showed that incorporation of silk and HA particles in the PLGA scaffolds could impart optimal hydrophilicity and stress-bearing capacity to the scaffolds along with more favorable bioactivity and biocompatibility to ameliorate greater cell infiltration and growth. Tian et al. (2021) attempted to fabricate EPL/PCL/HA hybrid scaffolds with EPL and found that compared to PCL and PCL/HA, EPL/PCL/HA reduced the infiltration of pro-inflammatory M1 macrophages, Th1, and Th17 and increased anti-inflammatory Th2 infiltration into the lesion area, showing stronger bone repair capacity. Xia et al. (2019) prepared PLGA/GM scaffolds. The addition of GMs improved the mechanical properties of PLGA and gave BMP-2 sustained release properties. BMP-2, which is a member of the TGF-β superfamily, can activate macrophages alone through the pSmad1/5/8 signaling pathway, promoting osteogenic differentiation of BMSCs and inducing osteogenesis by enhancing angiogenic factors to generate a positive feedback loop (Wei et al., 2018) and is currently widely used for bone regeneration in (Lieberman et al., 2002) defects that do not heal spontaneously. However, oral or injection administration of BMP-2 may result in systemic exposure and low local concentrations, so BMP-2 with PLGA/GM stents *in vivo* represent a promising therapeutic approach to treat bone defects.

### 4.5 Four-dimensional biomaterials

Bone regeneration occurs constantly during well-orchestrated process that requires participation and coordination of various components in a precise and sequential manner. With the development of 4D bioprinting technology, a variety of 3D biomaterials are integrated with the concept of time into dynamic 3D pattern biological structures. 4D bone biomaterials with the characteristics of shape memory [i.e., change their shapes under various stimuli (e.g., light, humidity, pressure, temperature, magnetic field, electric fields, and mechanical stimulation) meet the needs of personalized bone defect repair (Campbell et al., 2014). Over time, the functional transformation of printed cell-laden structures is also considered a feature of 4D bone biomaterials, which enhance the osteogenic differentiation. Particularly, some NIR/thermoresponsive 4D biomaterials were designed for bone regeneration, which provide potential solutions for bone tissue engineering.

**FIGURE 7:**
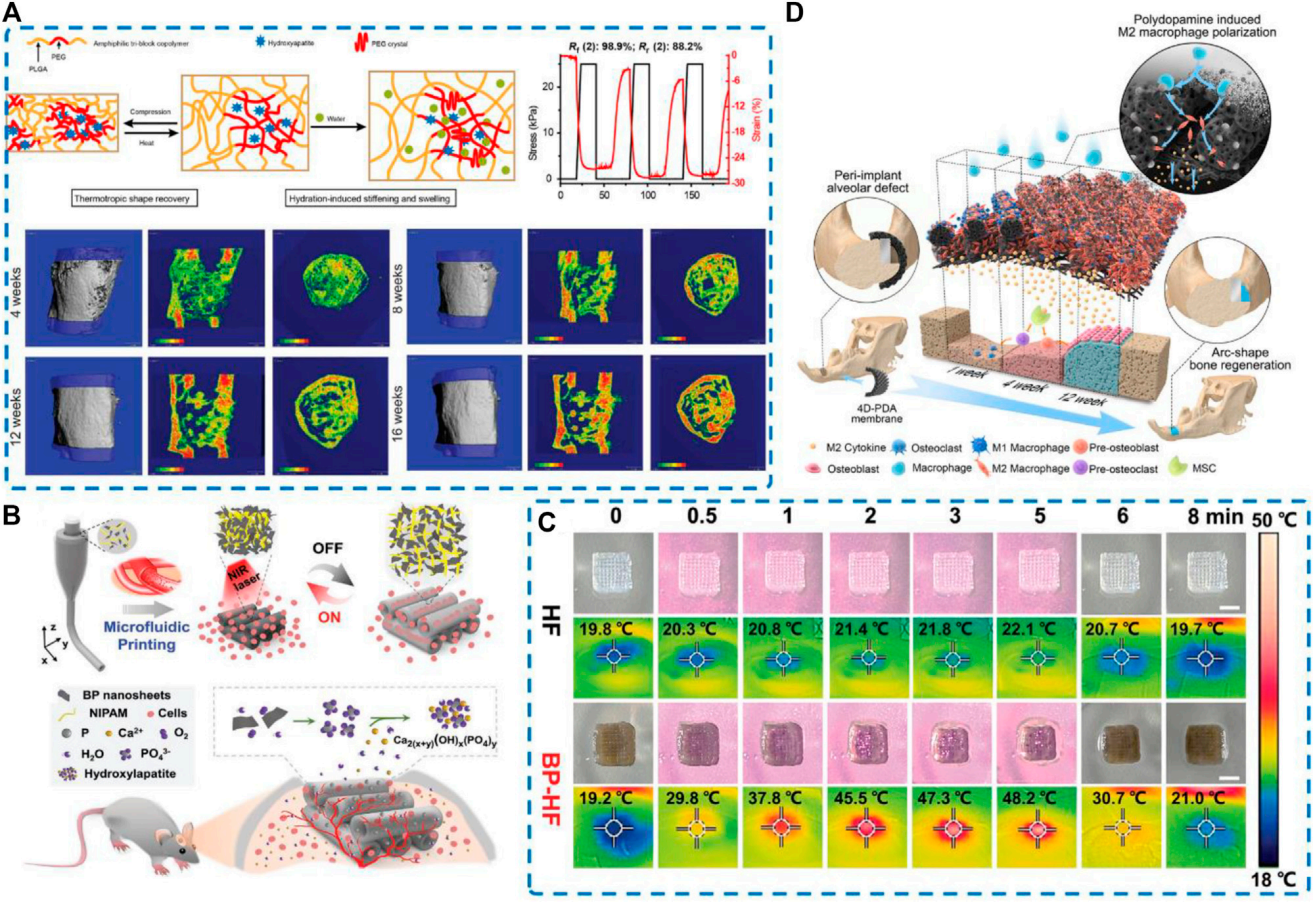
Schematic illustration and regenerative potential of four-dimensional biomaterials **(A)** 3D printing and characterization of the physical and thermomechanical properties of HA-PELGA composites; 3D μCT images and BMD color maps showing maturing regenerated bone within the defect over time. Reproduced from (Zhang et al., 2019b). **(B)** Schematic illustration of microfluidic 3D printing responsive scaffolds with biomimetic enrichment channels for bone regeneration. **(C)** Photothermal responsive performance of the BP-HF scaffolds. Real-time photograph and corresponding thermal images Reproduced from (Wang et al., 2022b) **(D)** Schematic diagram illustrating that the early and durable enrichment of M2 macrophages above the bone defect mediated by the 4D hierarchically channeled elastomeric membrane contributes to specific shape bone healing. Reproduced from (Liu et al., 2021b).

Zhang et al. (2019b) reported that 3D printing stimuli-responsive shape memory bone scaffolds are composed of HA and PELGA. Combined with a small quantity of rhBMP-2/7, hydration-induced swelling and stiffening behaviors of this 3D-printed graft scaffolds translated into more convenient implant placement and long-term success of implant-bone fixation, both critical for successful regeneration of segmental defects of the femur (Figure 7A). Liu et al. (2021b) found that the 4D-morphing of tough elastomer completely facilitate the regeneration of the dome shaped calvarial bone and arc-shape bone in periimplant alveolar defect, indicating that fabrication of 4D-morphing for PGS/PCL created a solution for reconstructing bone defects without additional agents or cells (Figure7D).

Present the same features as 4D material, 2D nanosheets present good biocompatibility, enhanced near-infrared (NIR) light absorption, and excellent photothermal conversion performance. Based on this, BP nanosheets can be endowed with functional transformation capability. Wang et al. proposed a microfluidic spinning 3D-printed strategy to endow the BP-incorporated fibrous scaffolds with repeated swelling/shrinkage behavior under action of NIR irradiation, which promotes cellular infiltration into the channels and facilitates the healing of bone defects (Wang et al., 2022b) (Figures 7B,C). In addition, NIR irradiation of the blood clot gel can accelerate bone regeneration *via* effective osteoimmunomodulation of microenvironment without any toxicity. Clot implantation and laser treatment can provide controlled regulation of the “immune niche” in the microenvironment of bone defects. Fan et al. (Fan et al., 2021) developed a platform loaded with BMP-2 and detected an increase in infiltrating macrophages in the BMP-2@BC-based osteoimplant on days 3 and 7. The infiltrating macrophages increased M1 polarization at the early stage of bone healing on days 3 and 7 but induced anti-inflammatory M2 phenotype at the lesion site on day 14. After modulating the immune niche, almost 95% of the defect area was covered by newly generated bone in disease models.

Despite advancements in achieving facile shape programming and shape recovery, the 4D biomaterials involve significant challenges in ensuring efficacy and safety (Montgomery et al., 2017). After implantation in coordination with the dynamic process of bone regeneration, 4D biomaterials provide spatiotemporal control of hierarchical microstructure and functionalities of fabricated substitutes.

## 5 Conclusion and perspectives

In recent years, BTE has been progressing rapidly, and new biomaterials and tissue engineering strategies have been developed to achieve osteogenic results better. In this review, we focus on the key role of the immune response in the skeletal system and the progress of bone biomaterials promoting osteogenesis by intervening in the bone immune microenvironment, such as altering the surface morphology or delivering different drugs, biological factors and trace element delivery. However, there are still some pressing issues that need to be addressed. Due to the chronological nature of the immune response, the best time for immunomodulation is the primary question. Meanwhile, bone regeneration is a dynamic process. Various injuries or different degrees of damage could lead to different healing effects in the clinic (Winkler et al., 2018), which presents challenges to the successful application of these bone materials. It also remains to be explored how to prolong the release and effects in and to develop novel strategies for cell delivery. In addition, most of the existing studies on immune response focus on the secretion of inflammatory cytokines or the transformation of macrophage phenotypes rather than the interaction of various parts in the whole immune microenvironment and its impact on osteogenesis and angiogenesis. Compared to macrophages, information about the role and potential of other immune cells, such as dendritic cells or T cells, in the bone regeneration is limited in the current studies. Another challenge is that only a few studies are reflected *in vivo* biomaterial-mediated osteoclastogenesis. We believe that improved strategies will help overcome them and open new avenues for the design of functionalized multidimensional biomaterials in bone regeneration.
